# Supplementary material for: Combining tubercidin and cordycepin scaffolds results in highly active candidates to treat late-stage sleeping sickness
Source: Nat Commun. 2019 Dec 5;10:5564. doi: 10.1038/s41467-019-13522-6 (PMC6895180; doi:10.1038/s41467-019-13522-6)
Supplement: Supplementary file 1 — Supplementary Information [file 41467_2019_13522_MOESM1_ESM.pdf]

Supplementary information

Hulpia et al., Combining tubercidin and cordycepin scaffolds results in highly active candidates to treat late-stage sleeping sickness

Supplementary Figure 1: Trypanocidal/trypanostatic effects of nucleoside analogues

A

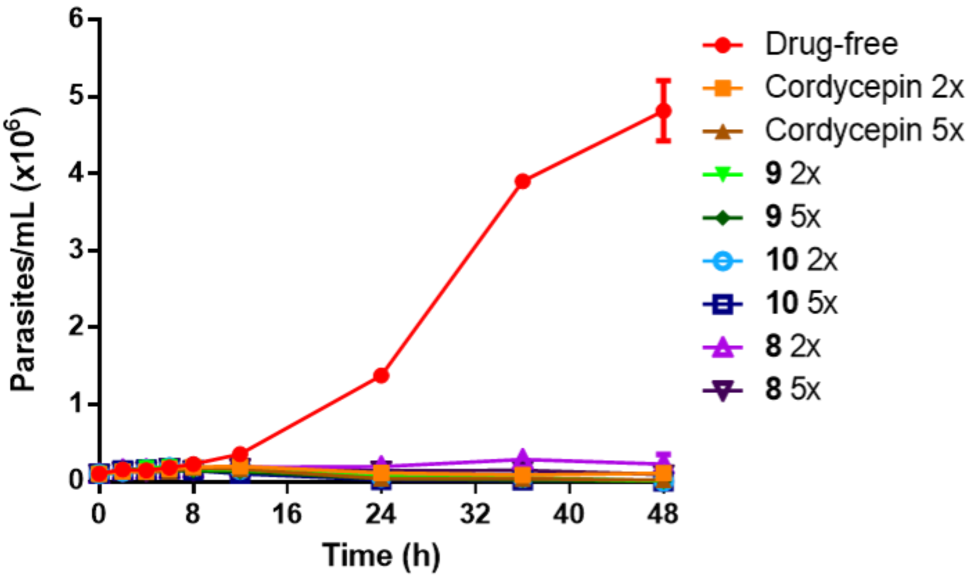

B

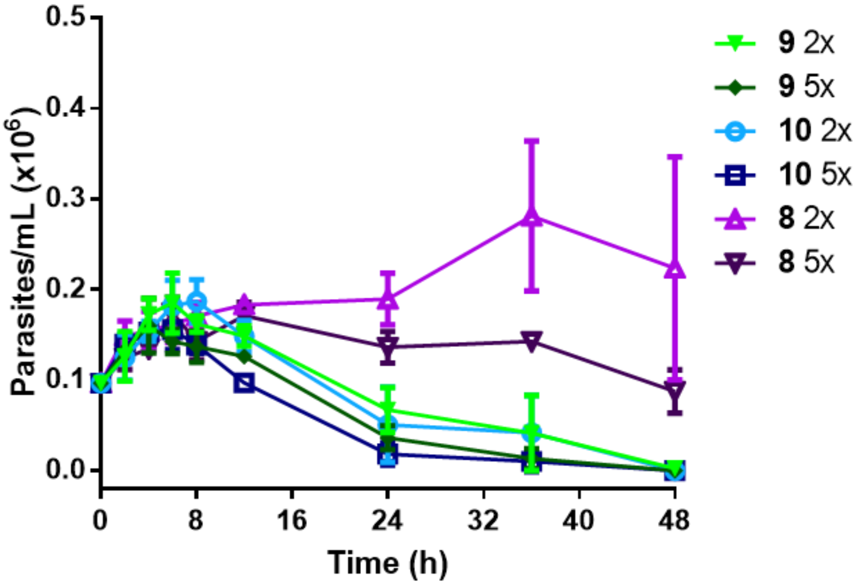

Supplementary Figure 1. Panel A. Proliferation of *T. brucei* Lister 427 wildtype bloodstream forms in culture in the presence of 2x and 5x EC_{50} of adenosine analogues. Panel B. Zoom in of panel A (base of the graph), showing the trypanocidal effect of **9** and **10** and the apparently trypanostatic effect of **8**. Results represent the mean ± SEM of two independent experiments (*n*=2) performed in duplicate, with four independent cell counts at each time point. Source data are provided as a Source Data file.

Supplementary Figure 2: Washout experiments with nucleoside analogue 9

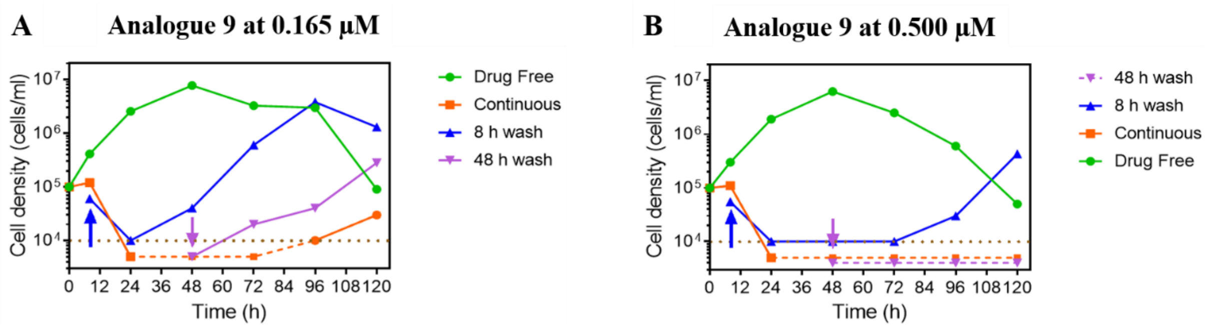

Supplementary Figure 2: Panel A. Manual cell count of *T. brucei* Lister 427 wildtype bloodstream forms in culture grown in the presence of absence of 0.165 µM (5xEC_{50}) of analogue **9**. Panel B. Manual cell count of *T. brucei* Lister 427 wildtype bloodstream forms in culture grown in the presence of absence of 0.500 µM (15xEC_{50}) of analogue **9**. The arrows (8 h and 48 h) indicate the time points of washout onset. Source data are provided as a Source Data file.

Supplementary Figure 3: Trypanosome cell morphology after incubation with nucleoside analogues

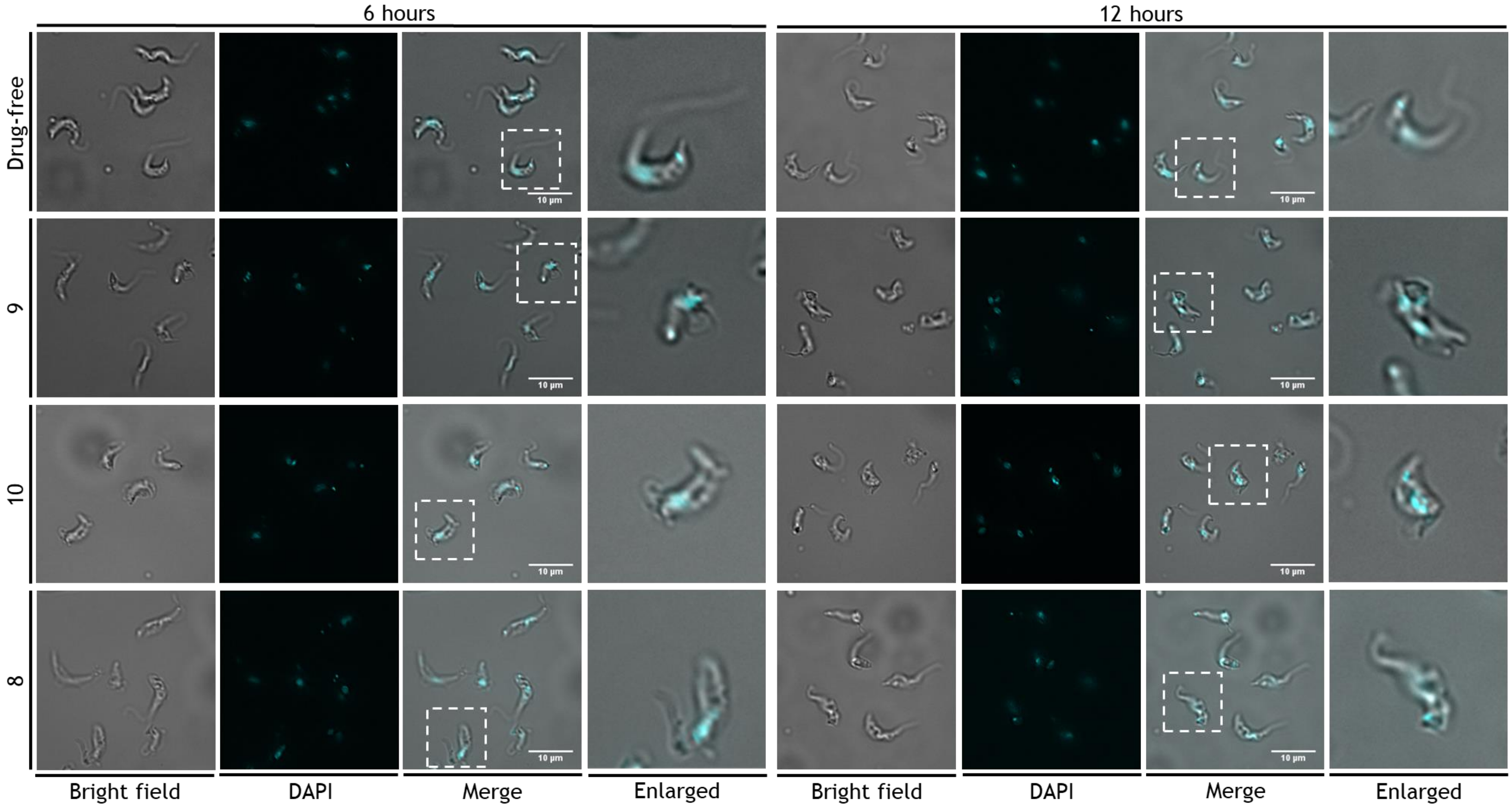

Supplementary Figure 3: Trypanosome cell morphology is altered after exposure to adenosine analogues at 5x EC_{50}. Cells were collected at 6 and 12 h of exposure to adenosine analogues, fixed and their morphology analysed by microscopy. A clearly altered morphology is observed in treated cells after 12 h

with distortion of the cell shape and apparent formation of intracellular vacuoles. DAPI - 4',6-diamino-2-phenylindole. Scale bars represent 10 μm. One representative cell of each group was enlarged for clearer visualisation.

Supplementary Figure 4: *In vivo* evaluation of analogue 9 in a mouse model of acute infection with *T. b. brucei*

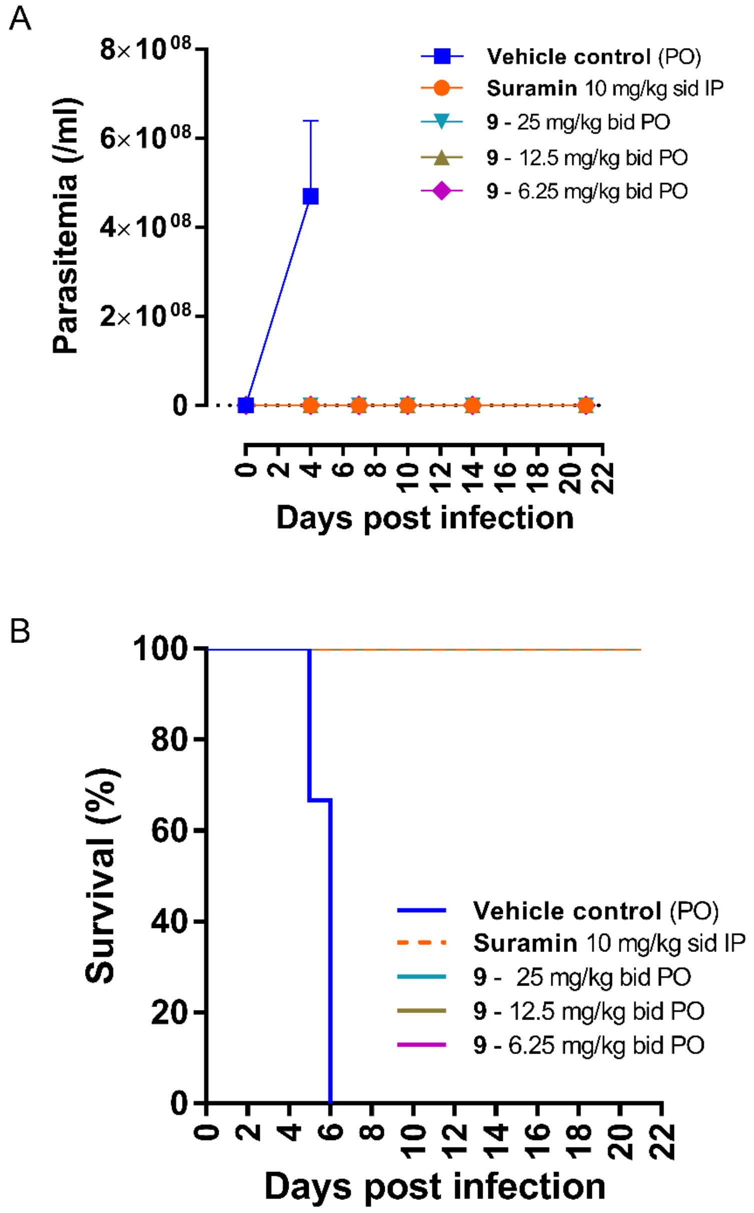

Supplementary Figure 4: Evaluation of the *in vivo* activity of nucleoside analogue **9** in the acute mouse model of HAT (*T. b. brucei*, Squib 427-suramin sensitive). Nucleoside analogue **9** was administered for 5 consecutive days, starting at the day of infection, in a dosing regimen as indicated for each treatment group (*n*=3 per treatment group). Panel A: Blood parasitaemia evaluation of tail vein blood. Panel B: Survival analysis. Source data are provided as a Source Data file.

Supplementary Figure 5: Dose-titration of analogue 9 in a stage-II model of HAT

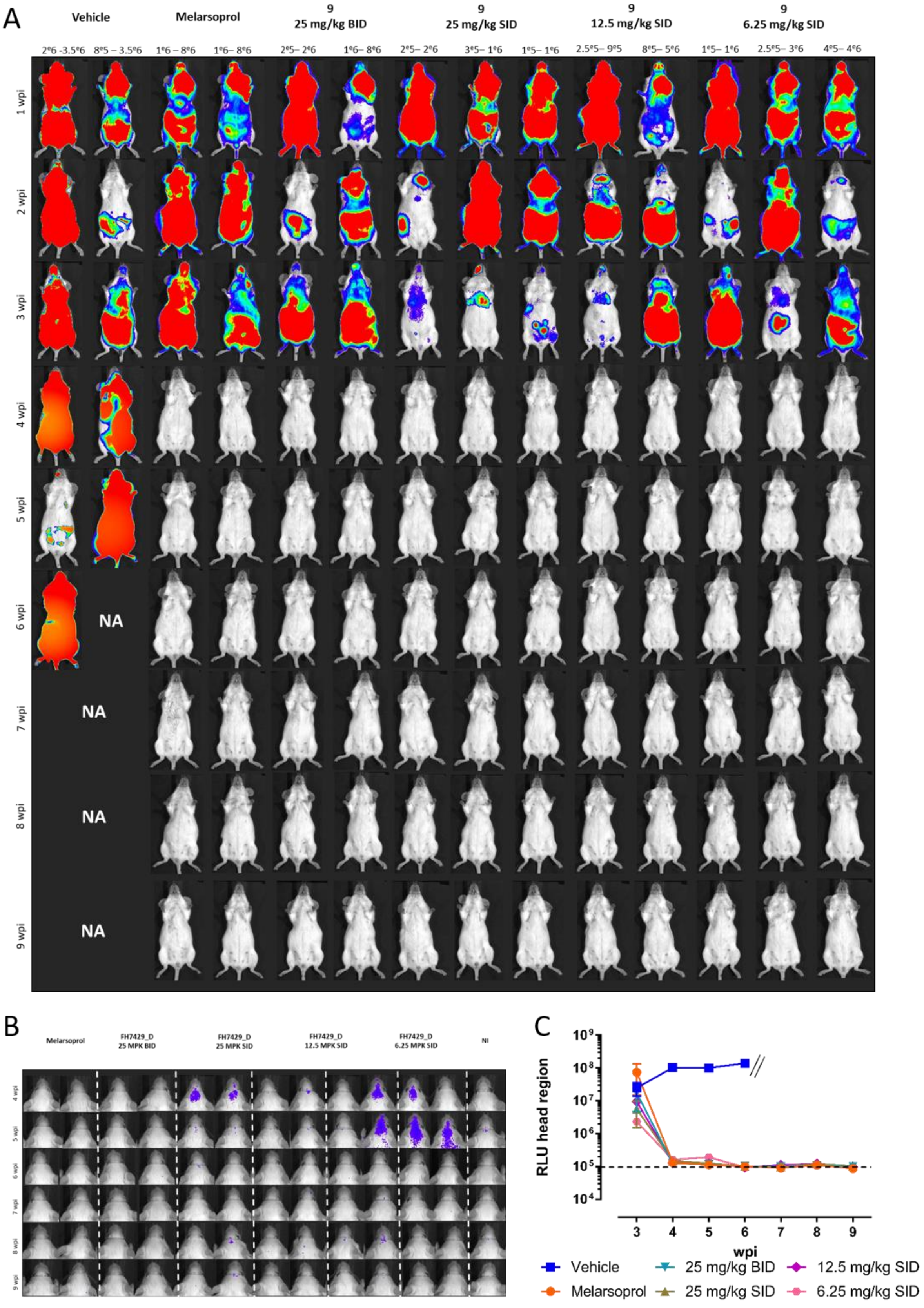

Supplementary Figure 5: *In vivo* evaluation of **9** in a stage II mouse model of HAT. (**A**) BLI images of stage II *T. b. brucei* (AnTAR1.1 PPYRE9) infected mice treated at 21 dpi (3wpi) with vehicle (*n*=2), melarsoprol (*n*=2) or **9** at different dose and dosing schedule, as indicated on the horizontal axis (25 mg/kg b.i.d. *n*=2; 25 mg/kg s.i.d. *n*=3; 12.5 mg/kg s.i.d. *n*=2; 6.25 mg/kg s.i.d. *n*=3). Images were acquired from the dorsal side of the animal. Image scales are indicated for each animal. (**B**) BLI images of the head region of melarsoprol and compound **9** treated mice, with a non-infected animal (NI) as a reference. (**C**) BLI (average signal ± SEM) of the head region. // = data not available due to animals in the control group succumbing to infection. Dashed lined represents the background signal in a non-infected animal. Source data are provided as a Source Data file.

Supplementary Figure 6: Drug sensitivity of RNAi clones

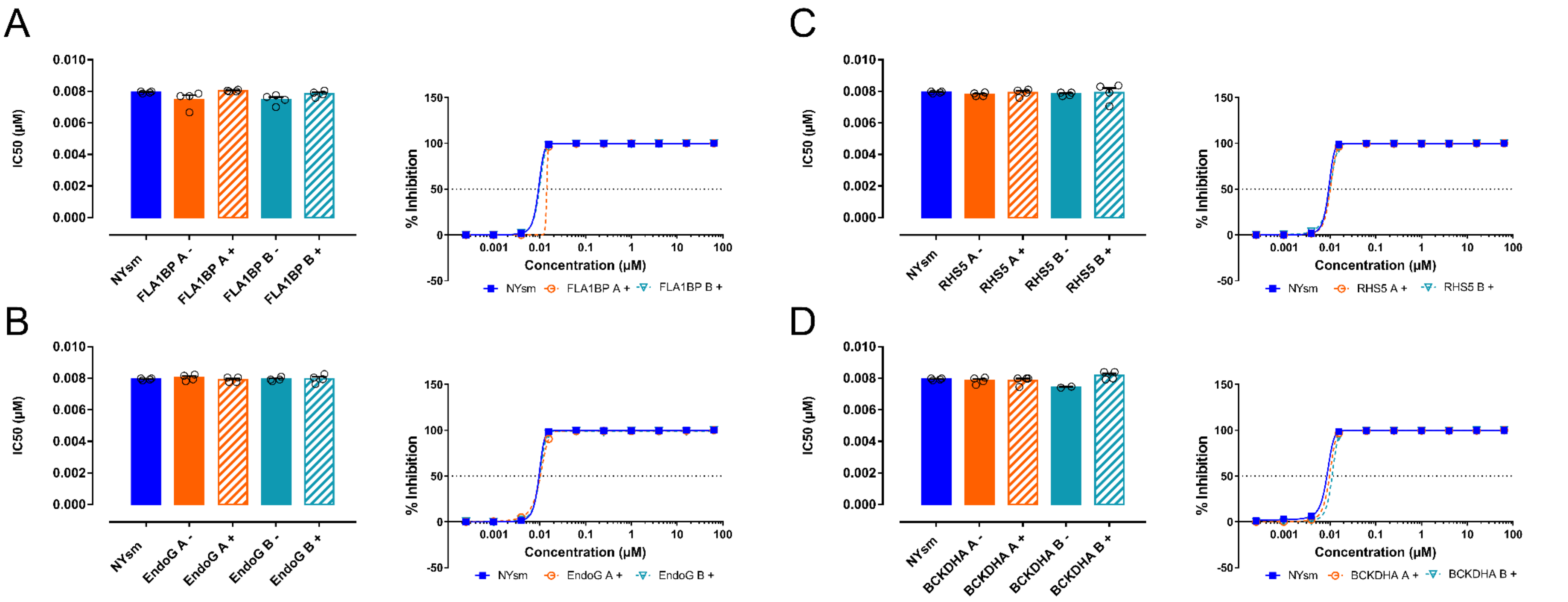

Supplementary Figure 6: Drug susceptibility of RNAi clones targeting (A) FLA1BP (FLA1 binding protein), (B) EndoG (Endonuclease G), (C) RHS5 (Retrotransposon Hotspotprotein 5), (D) BCKDHA (branched chain keto acid dehydrogenase, i.e. 2-oxoisovalerate dehydrogenase). Results are expressed as the mean IC_{50} (µM) ± SEM and are based on two independent experiments with two biological replicates. + = tetracycline-induced clones. - = non-induced clones. All experiments were performed with two independent RNAi clones (A and B). Source data are provided as a Source Data file.

Supplementary Figure 7: Nucleotide pool analysis – optimized conditions

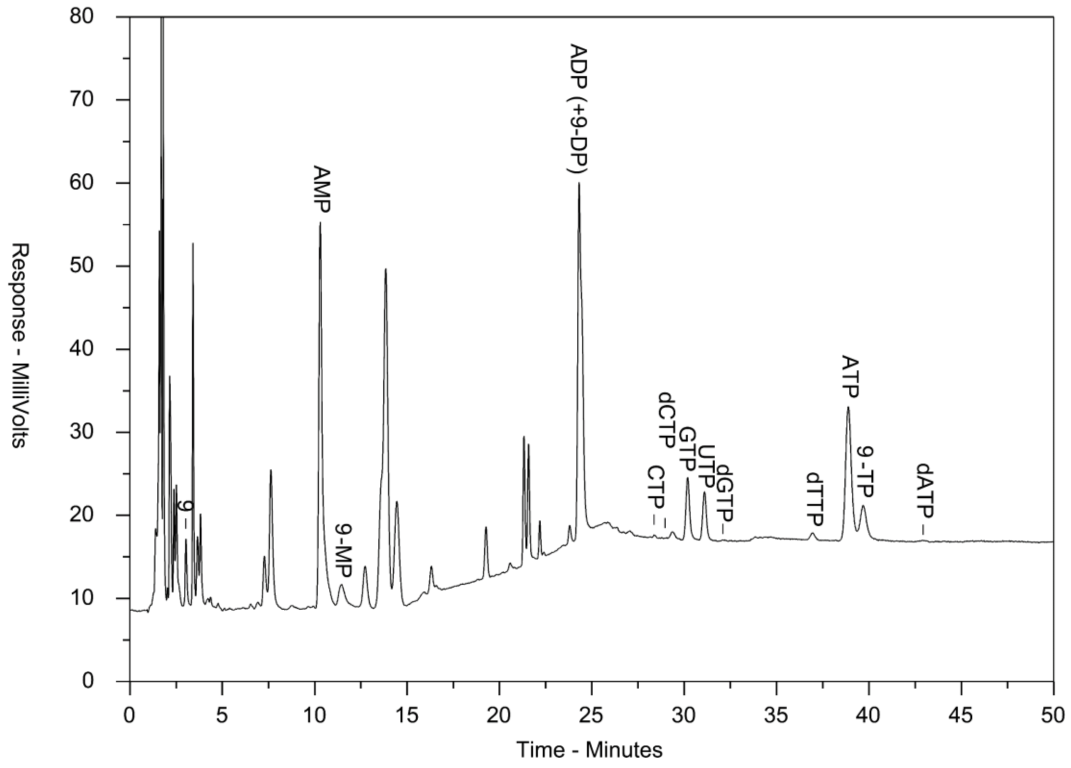

Chromatographic details:

Column: ACE Excel 2 μm C18, 150x2.1 mm.

Mobile phase:

Solvent A: 7% acetonitrile in aq. KH_2PO_4 (23 g L^{-1}) (pH adjusted with KOH; pH 6.3)

Solvent B: 7% acetonitrile in H_2O

Solvent C: 7% acetonitrile in aq. tetrabutylammonium bromide (3.52 g L^{-1})

Gradient: 4:76:20 of A:B:C isocratically for 11 min, gradient at 11-23 min to 27:53:20 of A:B:C, and isocratically 27:53:20 of A:B:C to 50 min.

Temperature: 52°C, loop size: 20 μL, detector 270 nm STD, flow rate 0.3 mL min^{-1}.

Sample: Extract from *T. brucei* cells incubated with nucleoside analogue **9**.

Supplementary Figure 8: Nucleotide pool analysis isocratic conditions – for separation of 9-diphosphate (9-DP) and 9-triphosphate (9-TP).

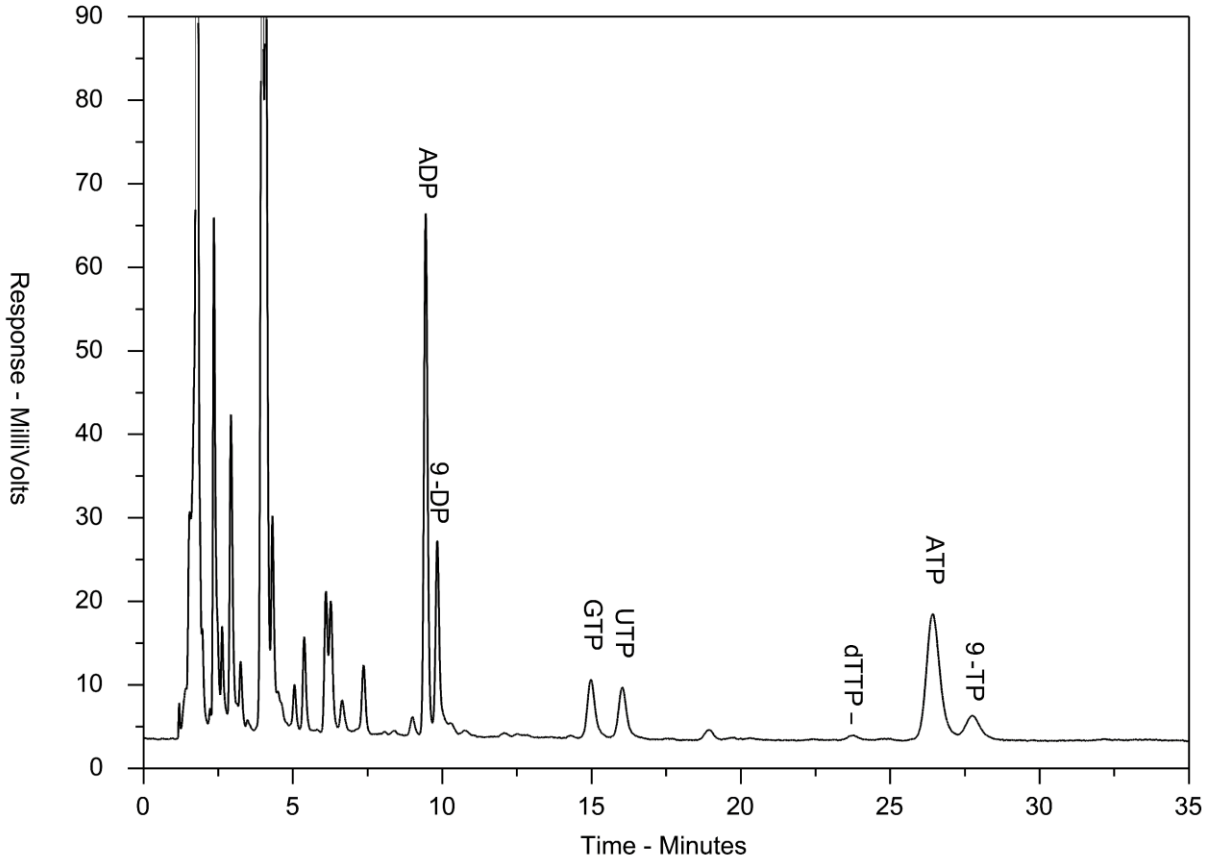

Chromatographic details:

Column: ACE Excel 2 µm C18, 150x2.1 mm.

Mobile phase:

Solvent A: 7% acetonitrile in aq. KH_2PO_4 (23 g L^{-1}) (pH adjusted with KOH; pH 6.3)

Solvent B: 7% acetonitrile in H_2O

Solvent C: 7% acetonitrile in aq. tetrabutylammonium bromide (3.52 g L^{-1})

Isocratic: 24:56:20 of A:B:C

Temperature: 55°C, loop size: 20 µL, detector: 270 nm STD, flow rate 0.27 mL min^{-1}.

Sample: Extract from *T. brucei* cells incubated with nucleoside analogue **9**.

Supplementary Figure 9: Nucleotide pool analysis - optimization of separation of analogue 9-MP

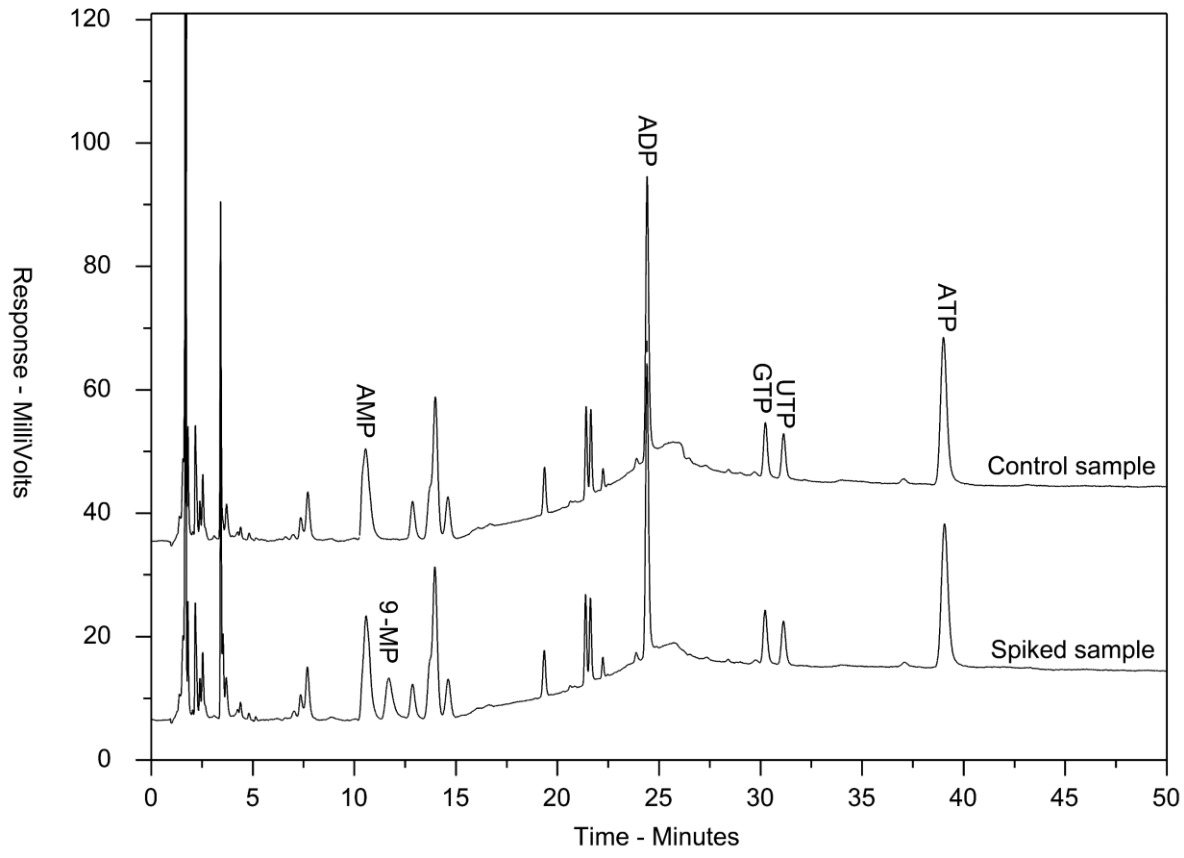

Chromatographic details:

Column: ACE Excel 2 µm C18, 150x2.1 mm.

Mobile phase:

Solvent A: 7% acetonitrile in aq. KH_2PO_4 (23 g L^{-1}) (pH adjusted with KOH; pH 6.3)

Solvent B: 7% acetonitrile in H_2O

Solvent C: 7% acetonitrile in aq. tetrabutylammonium bromide (3.5 g L^{-1})

Gradient: 4:76:20 of A:B:C isocratically for 11 min, gradient at 11-23 min to 27:53:20 of A:B:C, and isocratically 27:53:20 of A:B:C to 50 min.

Temperature: 52°C, loop size: 20 µL, detector 270 nm STD, flow rate 0.3 mL min^{-1}.

Sample: control *T. brucei* sample. The lower chromatogram has been spiked with the synthesized monophosphate (**9-MP**).

Supplementary Figure 10: Enzymatic preparation of triphosphate standard for HPLC analysis - conversion of 9 to the triphosphate form

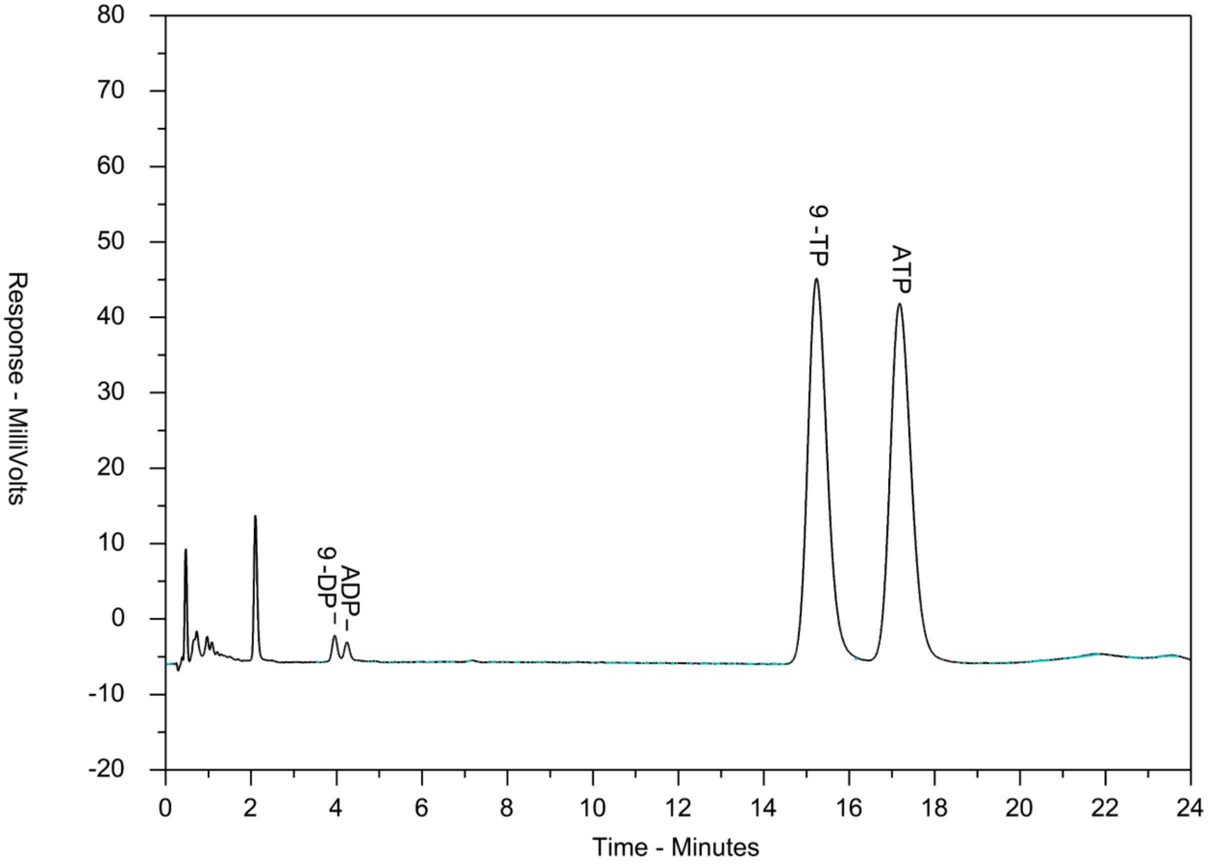

Chromatographic details:

<u>Column</u>: ACE Excel 2 µm C18, 50 x 3 mm.

<u>Mobile phase</u>:

Solvent A: 7% acetonitrile in aq. KH_2PO_4 (23 g L^{-1}) (pH adjusted with KOH; pH 6.3)

Solvent B: 7% acetonitrile in H_2O

Solvent C: 7% acetonitrile in aq. tetrabutylammonium bromide (3.52 g L^{-1})

<u>Isocratic</u>: 20:60:20 of A:B:C

<u>Temperature</u>: ambient, loop size: 20 µL, detector: 270 nm STD, flow rate 0.27 mL min^{-1}.

<u>Sample</u>: nucleoside **9** incubated in an enzymatic assay with adenosine kinase, adenylate kinase and creatine kinase to prepare an HPLC reference containing **9**-triphosphate (**9-TP**).

Supplementary Figure 11: Enzymatic preparation of standards for nucleotide pool analysis – conversion of 9 to mono-, di and triphosphates

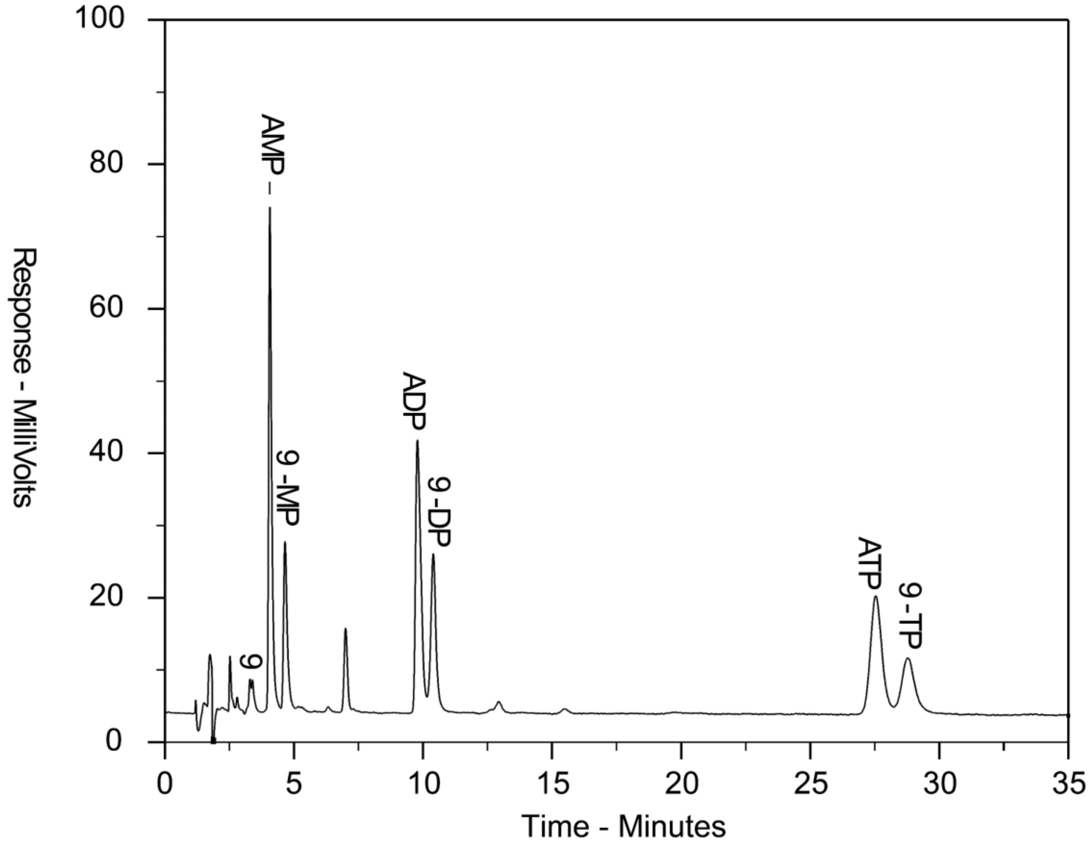

Chromatographic details:

Column: ACE Excel 2 µm C18, 150 x 2.1 mm.

Mobile phase:

Solvent A: 7% acetonitrile in aq. KH_2PO_4 (23 g L^{-1}) (pH adjusted with KOH; pH 6.3)

Solvent B: 7% acetonitrile in H_2O

Solvent C: 7% acetonitrile in aq. tetrabutylammonium bromide (3.52 g L^{-1})

Isocratic: 24:56:20 of A:B:C

Temperature: 50°C, loop size: 20 µL, detector: 270 nm STD, flow rate 0.27 mL min^{-1}.

Sample: nucleoside analogue **9** incubated in an enzymatic assay with adenosine kinase and adenylate kinase to prepare an HPLC reference containing **9**-nucleotides. The concentration of free nucleoside is negligible (and hidden in another peak), and therefore not used in the quantification of total **9** nucleosides/nucleotides.

Supplementary Figure 12: HPLC analysis of adenosine kinase assays

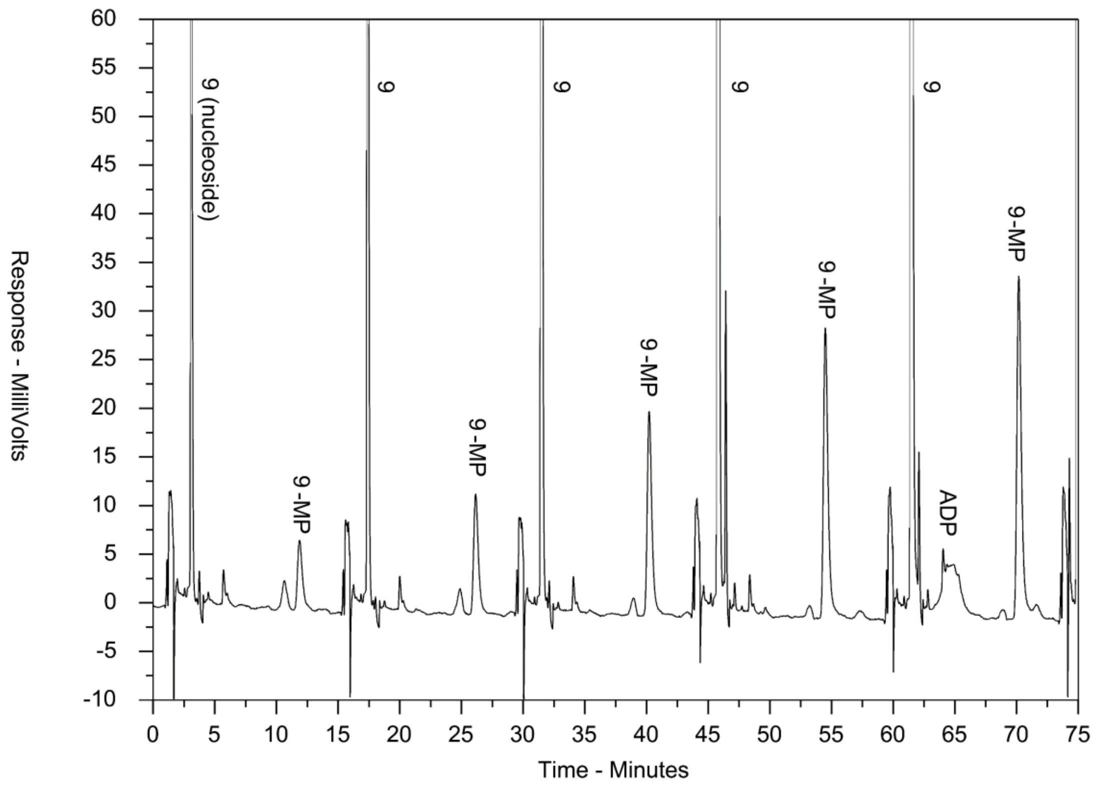

Chromatographic details:

Column: ACE Excel 2 µm C18, 150 x 2.1 mm.

Mobile phase:

Solvent A: 7% acetonitrile in aq. KH_2PO_4 (23 g L^{-1}) (pH adjusted with KOH; pH 6.3)

Solvent B: 7% acetonitrile in H_2O

Solvent C: 7% acetonitrile in aq. tetrabutylammonium bromide (3.52 g L^{-1})

Isocratic: 4:76:20 of A:B:C

Temperature: 52°C, loop size: 20 µL, detector: 270 nm STD, flow rate 0.3 mL min^{-1}.

Sample: Consecutive adenosine kinase assay samples loaded with ~15 min between each loading. The last sample above is deliberately loaded at a time point to not interfere with the ADP peak from the first sample coming at 65 min. After 4-5 runs, the column was washed with 80:20 of A:C to wash out ADP and ATP from all previous samples before returning to the original mobile phase composition.

Supplementary Table 1: Metabolic stability of nucleoside analogue 9

Phase I / II	Time	MOUSE % analogue 9 remaining		RAT % analogue 9 remaining		HUMAN % analogue 9 remaining	
		Mean	STDEV	Mean	STDEV	Mean	STDEV
CYP - NADPH	0	100	-	100	-	100	-
	15	110	6.6	68	4.0	107	14.8
	30	103	10.2	83	1.5	95	2.4
	60	111	10.7	76	0.5	102	7.8
UGT Enzymes	0	100	-	100	-	100	-
	15	100	2.3	99	15.9	115	6.5
	30	99	12.6	95	13.0	119	6.5
	60	110	10.0	119	13.5	106	20.1

Supplementary Table 1: Assessment of *in vitro* metabolic stability (phase I and phase II) of nucleoside analogue **9** using mouse, rat and human S9 microsomal fractions. The table represents the percentage of remaining parent compound, assayed at various time points of incubation (0-15-30-60 min). Two replicate measurements were performed. The *in vitro* assay was validated by employing diclofenac (susceptible to Phase-I and Phase-II metabolism) and fluconazole (metabolically stable through Phase-I) as reference compounds (*vide infra*).

Phase I / II	Time	MOUSE % parent compound remaining		RAT % parent compound remaining		HUMAN % parent compound remaining	
		Diclo	Fluco	Diclo	Fluco	Diclo	Fluco
CYP - NADPH	0	100	100	100	100	100	100
	15	83±14	111±12	59±9	110	35±0.4	110
	30	67±5.7	93±19	39±2.6	104	14±0.8	80
	60	52±0.3	100±12	10±2.9	96	2±0.4	103
UGT Enzymes	0	100	100	100	100	100	100
	15	42±21	99±19	28±4.5	111	17±1.1	103
	30	46±10	104±3	14±1.5	105	14±0.7	116
	60	34±9	100±6	11±0.9	112	10±0.1	106

Supplementary Table 2: Adenosine deaminase (ADA) susceptibility

	T. b. brucei **EC_{50} (µM)**	
**Cpd.**	-Dcf	+Dcf
**3, cordycepin**	0.44 ± 0.11	0.0036 ± 0.001*
**6, Tubercidin**	0.22 ± 0.01	0.31 ± 0.07
**9, 3'-deoxy-tubercidin**	0.037 ± 0.003	0.049 ± 0.009
**10, 3'-deoxy-7-Br-tubercidin**	0.0020 ± 0.0001	0.0019 ± 0.0002

Supplementary Table 2: Investigation of the susceptibility of selected nucleoside analogues for adenosine deaminase (ADA)-mediated breakdown. Drug sensitivity assays were performed either with Lister 427WT *T. b. brucei* in the absence (-Dcf) or presence of 2 µM deoxycoformycin (+Dcf), a known ADA inhibitor. Values represent the mean ± SEM of three independent experiments (*n*=3). Structures of assayed analogues are depicted below. * = p ≤ 0.05, student *t*-test (two-sided). Source data are provided as a Source Data file.

Supplementary Table 3: qPCR data of tissue samples from surviving mice

Target	Group	Sample	Tissue	Ct		Tm (cut-off 65.9-66.9)		Tissue	Ct		Tm (cut-off 65.9-66.9)		Tissue	Ct		Tm (cut-off 65.9-66.9)	
SL-RNA	Untreated	1	Brain	17.81	17.68	66.47	66.47	Fat	13.43	13.30	66.48	66.48	Spleen	14.89	14.89	66.18	66.18
	Melarsoprol	1		32.93	32.66	76.33	76.33		36.36	36.81	64.83	64.83		34.58	35.64	77.08	77.23
		2		35.25	32.98	76.48	76.18		u.d.	u.d.	u.d.	u.d.		u.d.	u.d.	u.d.	u.d.
	9 - 25 mg/kg b.i.d.	1		37.76	33.85	76.63	76.18		u.d.	36.15	u.d.	64.83		35.80	36.77	77.08	77.23
		2		34.59	34.22	76.33	76.18		u.d.	u.d.	u.d.	u.d.		37.57	36.16	77.23	77.08
	9 - 25 mg/kg s.i.d.	1		33.44	32.72	76.18	76.18		u.d.	u.d.	u.d.	u.d.		34.08	35.33	76.93	76.93
		2		33.49	32.91	76.33	76.18		u.d.	u.d.	u.d.	u.d.		37.55	34.50	77.23	77.08
		3		34.54	36.33	76.48	76.48		u.d.	34.47	u.d.	65.13		35.19	34.69	64.53	77.23
	9 - 12.5 mg/kg s.i.d.	1		32.69	31.87	76.48	76.48		35.64	u.d.	64.83	u.d.		u.d.	36.78	u.d.	77.08
		2		34.24	32.77	76.48	76.48		31.99	31.28	76.18	76.03		38.18	35.84	77.37	77.37
	9 - 6.25 mg/kg s.i.d.	1		33.20	32.28	76.33	76.48		u.d.	34.72	u.d.	65.13		34.96	34.51	77.08	76.63
		2		31.98	32.45	76.33	76.33		u.d.	u.d.	u.d.	u.d.		u.d.	36.65	u.d.	77.08
		3		32.18	32.22	76.18	76.18		u.d.	u.d.	u.d.	u.d.		35.20	33.81	76.93	76.78

Target	Group	Sample	Tissue	Ct		Tm (cut-off 86.9-87.9)		Tissue	Ct		Tm (cut-off 86.9-87.9)		Tissue	Ct		Tm (cut-off 86.9-87.9)	
Eef2	Untreated	1	Brain	14.73	15.20	87.52	87.52	Fat	15.23	15.32	87.52	87.37	Spleen	13.79	13.51	87.36	87.36
	Melarsoprol	1		15.26	14.90	87.52	87.52		15.58	15.67	87.37	87.37		14.54	15.17	87.52	87.52
		2		14.46	14.59	87.52	87.37		17.44	16.79	87.22	87.22		14.94	15.15	87.37	87.37
	9 - 25 mg/kg b.i.d.	1		15.86	15.93	87.37	87.37		17.17	17.12	87.22	87.22		16.82	16.93	87.37	87.37
		2		14.65	14.27	87.37	87.37		17.76	17.75	87.22	87.22		15.84	15.72	87.37	87.37
	9 - 25 mg/kg s.i.d.	1		14.59	14.99	87.37	87.37		17.00	16.97	87.22	87.22		15.59	15.41	87.37	87.37
		2		14.82	15.20	87.37	87.37		20.32	20.18	87.22	87.22		15.13	15.37	87.37	87.37
		3		14.93	14.87	87.52	87.52		17.28	17.29	87.37	87.37		16.45	16.68	87.52	87.52
	9 - 12.5 mg/kg s.i.d.	1		15.24	14.77	87.67	87.67		16.33	16.54	87.37	87.52		15.40	15.64	87.36	87.36
		2		14.92	15.20	87.66	87.52		14.65	14.57	87.37	87.37		14.96	15.34	87.52	87.67
	9 - 6.25 mg/kg s.i.d.	1		14.69	14.83	87.67	87.67		17.72	17.79	87.37	87.37		16.81	16.74	87.22	87.22
		2		14.91	14.67	87.67	87.67		17.85	17.92	87.37	87.37		15.38	15.53	87.36	87.36
		3		14.93	14.85	87.52	87.52		17.43	17.35	87.37	87.37		15.49	15.31	87.22	87.36

Supplementary Table 3: qPCR analysis of tissue samples (brain, fat and spleen) from surviving animals to probe for potential residual parasite levels. Cells in the table that are coloured in green are positive for the specific amplification product (SL-RNA in the case of *T. brucei* (upper part) or the mouse reference gene

Eef2 (lower part)). Cells coloured in red are negative for the specific amplification product. Upper part: tissue samples tested for the presence of SL-RNA by qPCR, probing for the presence of *T. brucei* parasites. Lower part: control samples tested for the presence of the mouse reference gene Eef2, demonstrating appropriate RNA extraction efficiency in all tested samples. Tissue samples originate from the animal experiment depicted in Supplementary Figure S5. u.d. = undetected.

Supplementary Table 4: Overview of sequencing results of RNAi inserts

Primer	Gene	Gene Product	Annotation	Location RNAi	RNAi match
M13-F	/	/	/	/	/
M13-R	Tb927.6.5830	variant surface glycoprotein, degenerate	Tb927_06_v5.1:1614636-1616066(-)	1-816	747/820
M13-F	Tb927.8.4050	FLA1-binding protein	Tb927_08_v5.1:1203333-1205585(-)	2279-3027	738/749
M13-R	Tb927.8.4040	endonuclease G, putative	Tb927_08_v5.1:1200841-1202361(-)	1-389	344/347
M13-F	Tbg972.5.40	adenosine transporter	Tbg972_05:6796-8187(-)	175-1307	1096/1135
M13-R	Tbg972.5.40	adenosine transporter	Tbg972_05:6796-8187(-)	378-1392	997/1016
M13-F	Tb927.2.980	retrotransposon hot spot protein 5	Tb927_02_v5.1:167874-168341(+)	5856-6792	867/943
M13-R	Tb927.2.980	retrotransposon hot spot protein 5	Tb927_02_v5.1:167874-168341(+)	5854-6793	872/947
M13-F	Tb11.v5.1012	2-oxoisovalerate dehydrogenase beta subunit, mitochondrial precursor putative	Tb927_11_bin_v5.1:5313959-5315065(-)	1-571	569/571
M13-R	Tb11.v5.1012	2-oxoisovalerate dehydrogenase beta subunit, mitochondrial precursor putative	Tb927_11_bin_v5.1:5313959-5315065(-)	1-571	569/571
M13-F	Tb927.6.2300	adenosine kinase, putative	Tb927_06_v5.1:718416-719453(+)	1-317	316/317
M13-R	Tb927.6.2300	adenosine kinase, putative	Tb927_06_v5.1:718416-719453(+)	1-317	316/317
M13-F	Tb927.4.5500	variant surface glycoprotein, degenerate	Tb927_04_v5.1:1501217-1502767(-)	810-1100	262/291
M13-R	Tb927.4.5500	variant surface glycoprotein, degenerate	Tb927_04_v5.1:1501217-1502767(-)	810-1100	262/291
M13-F	Tb927.6.2300	adenosine kinase, putative	Tb927_06_v5.1:718416-719453(+)	1-317	316/317
M13-R	Tb927.6.2300	adenosine kinase, putative	Tb927_06_v5.1:718416-719453(+)	1-317	316/317
M13-F	Tbg972.5.40	adenosine transporter	Tbg972_05:6796-8187(-)	384-1392	964/1012
M13-R	Tbg972.5.40	adenosine transporter	Tbg972_05:6796-8187(-)	175-1295	1063/1085
M13-F	Tb927.5.286b	adenosine transporter 1	Tb927_05_v5.1:87259-88650(-)	1300-2374	1027/1091
M13-R	Tb927.5.286b	adenosine transporter 1	Tb927_05_v5.1:87259-88650(-)	1300-2411	1052/1127

Supplementary Table 4: Overview of the sequencing results of individual RNAi inserts obtained following selection of a genome-wide *T. b. brucei* RNAi library exposed to **9**. M13-F = forward primer. M13-R = reverse primer.

Supplementary methods

Growth assays of *T. brucei*

To test the effect of the novel adenosine analogues on the growth of *T. brucei* BSF, s427 wildtype cells were seeded at 1×10^5 cells mL^{-1} in 5 mL of HMI-9 medium with 10% FBS and treated with compounds **8**, **9** and **10** and cordycepin at 2× and 5× the EC_{50} as determined by the Alamar blue assays. As control, cells were also incubated in drug-free medium under the same conditions. 2 µM of 2'-deoxycoformycin were added to all cultures to avoid any potential effect of serum adenosine deaminase on the activity of test compounds and cordycepin.[1]

Cells were manually counted in Neubauer chambers at 0, 2, 4, 6, 8, 12, 24, 36 and 48 h after addition of the drugs. At 6 and 12 h, cells were also collected and fixed to have their structure checked by microscopy.

Wash-out experiments with *T. brucei*

A culture of bloodstream form *T. brucei* 427WT, at mid-log phase of growth, was harvested and resuspended at a density of 10^5 cells mL^{-1}, in fresh medium containing either no test compound, or $5\times EC_{50}$ (165 nM; exp. 1) or 0.5 µM (exp. 2) of analogue **9**. The cultures were incubated under standard conditions (37 ºC/5% CO_2) and cell counts were performed manually using a haemocytometer under phase contrast microscopy, every 24 h. The culture in continuous presence of analogue **9** was split in two at 8 h and 48 h (as indicated with arrows in Supplementary Figure S2). The split cultures were centrifuged and resuspended in the same volume of either fresh medium without test compound or fresh medium with the same concentration of compound **9** as before, reincubated. The detection limit for the manual cell count was 10^4 cells mL^{-1}.

Microscopy

After 6 and 12 h after addition of drugs, approximately 2×10^6 cells were harvested from each group and applied to glass slides pre-treated with poly-L-lysine, left to settle for 5 min and fixed with 4% formaldehyde for 4 min. The formaldehyde was neutralised by addition of 100 mM glycine (20 min). Cells were washed once with PBS and blocked for 1 h with 1% BSA/0.2% Tween-20 in PBS. Cells were

washed thrice with PBS and 5 µL of 4',6-diamino-2-phenylindole (DAPI; SouthernBiotech) was added to each well and incubated for 4 min at room temperature in the dark. A coverslip was then placed on top of the wells and sealed with nail varnish.

Metabolic stability of nucleoside analogue 9

Mouse, rat, and pooled human liver microsomes were purchased from a commercial source (Corning) and stored at -80 °C. NADPH generating system solutions A and B and UGT reaction mix solutions A and B (Corning) were kept at -20°C. The test compound and the reference compounds diclofenac (MW 296.15) and fluconazole (MW 306.27) were formulated in DMSO at a concentration of 10 mM. The microsomal stability assay was carried out based on the BD Biosciences Guidelines for Use (TF000017 Rev1.0) with minor adaptations. Metabolic stability of the compounds was studied through the CYP450 superfamily (phase I metabolism) by fortification with reduced nicotinamide adenine dinucleotide phosphate (NADPH) and through uridine glucuronosyl-transferase (UGT) enzymes (phase II metabolism) by fortification with uridine diphosphate glucuronic acid (UDPGA). For the CYP450 and other NADPH dependent enzymes, both compounds were incubated at 5 µM together with 0.5 mg mL^{-1} liver microsomes in potassium phosphate buffer in a reaction started by the addition of 1 mM NADPH and stopped at 0, 15, 30 and 60 minutes. At these time points, 20 µL was withdrawn from the reaction mixture and 80 µL cold acetonitrile (ACN) containing the internal standard tolbutamide, was added to inactivate the enzymes and precipitate the proteins. The mixture was vortexed for 30 s and centrifuged at 4 °C for 5 min at 15,000 rpm. The supernatant was stored at -80°C until analysis. For the UGT enzymes, both compounds were incubated at 5 µM together with 0.5 mg mL^{-1} liver microsomes in a reaction started by the addition of 2 mM UDPGA cofactor. The corresponding loss of parent compound was determined using liquid chromatography (UPLC) (Waters Aquity™) coupled with tandem quadrupole mass spectrometry (MS^2) (Waters Xevo™), equipped with an electrospray ionization (ESI) interface and operated in multiple reaction monitoring (MRM) mode.

Adenosine deaminase susceptibility

The susceptibility of the nucleoside analogues towards adenosine deaminase-mediated breakdown was assessed by means of determining differences between drug sensitivity on *T. b. brucei* (Lister 427WT)

in the presence or absence of 2 μM 2'-deoxycoformycin. The drug sensitivity assay is described in the manuscript.

Nucleotide pool analysis

The HPLC equipment included three pumps (Smartline 1000, Knauer, Berlin, Germany) connected to a Standard mixer (Agilent Technologies, Santa Clara, CA, USA), a Keystone Hot Pocket 150 mm column heater (Thermo Fischer Scientific, Waltham, MA, USA), and a UV-2075 Plus detector (Jasco Corporation, Tokyo, Japan). The samples were analysed on a 2.1 x 150 mm ACE Excel (2 µm) C18 column (Advanced Chromatography Technologies, Aberdeen, UK) with a 2.1 ID ACE C4 (3 µm) guard column. The column temperature was set at 52°C, the flow rate was 0.3 mL min^{-1} and the detector wavelength was 270 nm (STD setting).

A gradient of three aqueous solutions was used: solution A (23 g L^{-1} KH_2PO_4 in 7% acetonitrile adjusted to pH 6.3 with KOH), solution B (7% acetonitrile) and solution C (3.52 g L^{-1} tetrabutylammonium bromide in 7% acetonitrile). The gradient started isocratically with a mixture of 4A:76B:20C over 11 min, followed by a gradient up to 27A:53B:20C between 11-23 min, and finally isocratic conditions at 27A:53B:20C between 23-50 min before returning to starting conditions. The column was re-equilibrated for 10 min between each run.

Using these conditions, it was possible to measure all NTPs and dNTPs (Supplementary Figure S9) as well as **9**-monophosphate (**9-MP**) and triphosphate (**9-TP**) (Supplementary Figure S7, and spiking experiment in Supplementary Figure S9). However, the separation between 9-diphosphate (**9-DP**) and ADP was not good, therefore, the quantification of these molecules were made using isocratic conditions with a mobile phase consisting of 24A:56B:20C (Supplementary Figure S8). The quantification of ATP was then used as a reference to be able to be able to merge the two data sets in Supplementary Figure S7 and S8. Generally, the peak height of each nucleotide was compared to the peak height in a standard nucleotide solution containing all NTPs and dNTPs for quantification.

Nucleotide standards for the **9**-di-and triphosphates (**9-DP** and **9-TP**, respectively), were created enzymatically prior to the analysis. The nucleoside **9** (100 µM) was then incubated for 30 min at 37°C

with 1 µg *T. brucei* adenosine kinase, 10 µg *E. coli* adenylate kinase and 6.25 µg rabbit skeletal muscle creatine kinase (Merck Group, Darmstadt, Germany) in a 50-µl reaction mixture containing 100 µM ATP, 1 mM $MgCl_2$, 20 mM phosphocreatine and 25 mM Tris-HCl pH 7.6. A nearly complete conversion to **9**-triphosphate (**9-TP**) was then achieved (Supplementary Figure S10). The sample was consequently centrifuged through a Nanosep 3k Omega filter to remove the enzymes. The addition of creatine kinase pushes the equilibrium of the nucleotides to **9**-triphosphate and ATP. By omitting creatine kinase, a mixture of **9** mono-, di- and triphosphates (as well as AMP, ADP and ATP) was obtained instead (Supplementary Figure S11). This mixture was then used as a reference to quantify the **9**-diphosphate (**9-DP**). The concentration of the **9**-diphosphate (**9-DP**) in this reference was then calculated as the initial nucleoside **9** concentration multiplied by the fraction of the diphosphate in the sample. The fraction of the diphosphate was obtained by taking the area of the diphosphate peak divided by the total area of all **9**-nucleotide peaks (*i.e.* mono-, di- and triphosphate).

Primers

The following primers were used:

Name	Sequence	Tm (°C)
SL-tr_F	AACTAACGCTATTATTAGAA	43.4
SL-tr_R	CAATATAGTACAGAAACTG	42.1
TERT_F	GAGCGTGTGACTTCCGAAGG	58.1
TERT_R	AGGAACTGTCACGGAGTTTGC	57.6
Eef2_F	TGTCAGTCATCGCCCATGTG	57.6
Eef2_R	CATCCTTGCGAGTGTCAGTGA	57.1
18S_tryp_F	ACGGAATGGCACCACAAGAC	58.1
18S_tryp_R	GTCCGTTGACGGAATCAACC	56.2
p2T7_seq	CCGCTCTAGAACTAGTGGA	52,9
p2T7hygPJ4	GGAAAGCTAGCTTGCATGCCTG	58,9
p2T7linker_rev	AGGGCCAGTGAGGCCTCTAGAG	62,4

Synthesis

All reagents and solvents were obtained from standard commercial sources and were of analytical grade. Unless specified otherwise, they were used without further purification. Tubercidin (**6**)[2] and **8**[3] were prepared as described in literature. 2'-Deoxytubercidin (**11**) was purchased from Carbosynth (Comptom, UK) and the identity and purity confirmed by means of ^{1}H-NMR[4] and LC/MS analysis.

All moisture sensitive reactions were carried out under an argon atmosphere. Reactions were performed at ambient temperature, unless otherwise indicated. Analytical TLC made use of precoated F254 Machery-Nagel® aluminum plates and were first investigated by UV followed by developing with basic aq. $KMnO_4$ staining or sulfuric acid-anisaldehyde spray. Column chromatography was performed using Machery-Nagel 60M silica gel (40-63 µm) or made use of a Reveleris X2 (Büchi) automated flash unit with pre-packed silica columns.

Exact mass measurements were performed on a Waters LCT Premier XE™ Time of Flight (ToF) mass spectrometer equipped with a standard electrospray (ESI) and modular Lockspray™ interface. Samples were infused in a MeCN/water (1:1) + 0.1 % formic acid mixture at 100 µL min^{-1}. NMR spectra are recorded on a Varian Mercury 300 MHz spectrometer. Chemical shifts (δ) are given in ppm and spectra are referenced to the residual solvent peak. Coupling constants are given in Hz. In ^{31}P NMR, signals are referenced to the DMSO lock resonance frequency according to IUPAC referencing, with H_3PO_4 set to δ=0.00 ppm.

Assignment of NMR peaks was verified by means of 2D NMR spectroscopic techniques: gCOSY, ^{1}H-^{13}C gHSQC, ^{1}H-^{13}C gHMBC whenever appropriate. Copies of relevant NMR spectra are included in the final section of this Supplementary Information. Melting points were determined on a Büchi-545 apparatus and are uncorrected.

Compound purity was assessed by means of analytical LC-MS employing a Waters AutoPurification system (equipped with ACQUITY QDa (mass; 100–1000 amu) and 2998 Photodiode Array (220–400 nm)). A Waters Cortecs® C18 (2.7 µm 100x4.6mm) column was used and the chromatographic

conditions consisted of a gradient of HCOOH in H_2O (0.2 %, v/v) / MeCN (95:05 to 00:100) in 6.5 minutes at a flow rate of 1.44 mL min^{-1}.

All obtained final compounds had purity >95 %, as assayed by analytical HPLC (UV).

Synthetic schemes

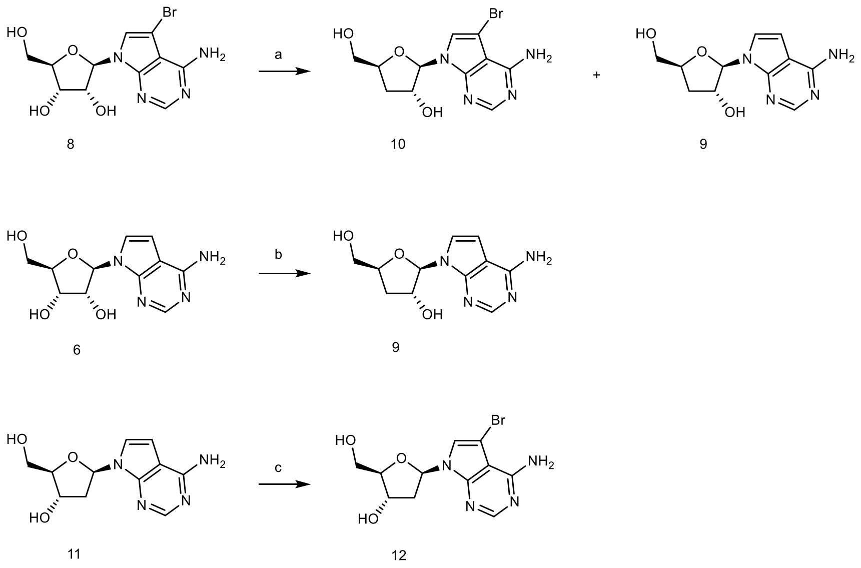

Supplementary Scheme 1: Reagents and conditions: a) 1. α-acetoxy-isobutyrylchloride, NaI, MeCN; 2. Pd/C, H_2 (balloon), aq. NaOAc, EtOH, overnight; 3. NH_3 7N in MeOH, rt., 49 % (**10**), 16 % (**9**); b) 1. α-acetoxy-isobutyrylchloride, NaI, MeCN; 2. Pd/C, H_2 (balloon), aq. NaOAc, EtOH, overnight; 3. NH_3 7N in MeOH, rt.; 43 %. c) NBS, DMF, 30 min, 21 %.

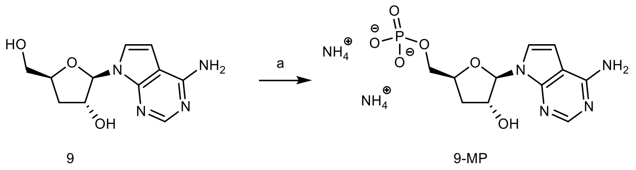

Supplementary Scheme 2: Reagents and conditions: a) 1. $POCl_3$, triethylphosphate, 0 °C, 2. 0.5 M aq. NH_4HCO_3, 46 %.

Experimental data

4-amino-5-bromo-N7-(3'-deoxy-β-D-ribofuranosyl)-pyrrolo[2,3-*d*]pyrimidine (10) (adapted from reference [5]) NaI (10 eq.) was dissolved in anhydrous MeCN (10mL/mmol SM) and stirred for 5 min under argon. Next, α-acetoxyisobutyrylchloride (3.5 eq.) was added, giving a white precipitate. The mixture was stirred vigorously for another 5–10 min, after which **8**[3] (0.35 g, 1.0 mmol, 1 eq.) was added in one portion. The resulting mixture was stirred for 1.5 h after which TLC showed full conversion of starting material. The mixture was poured in aq. sat. $NaHCO_3$/aq. sat. $Na_2S_2O_3$ solution. Next, $CHCl_3$ was added, and the layers separated. The water layer was extracted with $CHCl_3$ twice more. Organic layers were combined, dried over Na_2SO_4, filtered and evaporated. The resulting oil was dissolved in EtOH (7.5 mL/mmol SM) and 1M aq. NaOAc solution (2.5 mL/mmol SM) was added. Next, the flask was purged with N_2, after which a cat. amount of Pd/C was added. Next, the N_2-atmosphere was exchanged for H_2 (balloon; no bubbling) and the mixture stirred overnight. Then, the mixture was purged with N_2 to remover residual H_2-gas and filtered over a pad of Celite®. The mixture was evaporated till dryness and partitioned between EA and aq. sat. $NaHCO_3$/aq. sat. $Na_2S_2O_3$ solution. Layers were separated, and the water layer extracted twice more with EA. Organic layers were combined, dried over Na_2SO_4, filtered and evaporated. The resulting oil was dissolved in 7N NH_3 in MeOH and stirred overnight. The solvent was removed, and the residue purified by column chromatography 0 → 15 % MeOH/DCM, which gave **10** (0.16 g, 0.49 mmol) in 49 % yield as a white solid. Additionally, also **9** was isolated (0.041 g, 0.164 mmol) in 16 % yield.

Alternatively, **10** was prepared by means of glycosylation, as described.[6]

Analytical data for **10**:

Melting point: 223 °C.

^{1}H NMR (300 MHz, DMSO-d$_6$) δ: 1.87 (ddd, *J* = 13.2, 6.3, 3.3 Hz, 1H, H-3''), 2.14 – 2.23 (m, 1H, H-3'), 3.50 (ddd, *J* = 12.0, 5.4, 4.2 Hz, 1H, H-5''), 3.66 (ddd, *J* = 12.0, 5.4, 3.3 Hz, 1H, H-5'), 4.24 – 4.31 (m, 1H, H-4'), 4.34 – 4.39 (m, 1H, H-2'), 5.03 (t, *J* = 5.7 Hz, 1H, OH-5'), 5.57 (d, *J* = 4.5 Hz, 1H, OH-2'), 6.04 (d, *J* = 2.7 Hz, 1H, H-1'), 6.76 (br. s, 2H, NH_2), 7.65 (s, 1H, H-6), 8.11 (s, 1H, H-2). ^{13}C NMR

(75 MHz, DMSO-d_6) δ: 34.3 (C-3’), 62.5 (C-5’), 75.0 (C-2’), 80.1 (C-4’), 86.3 (C-5), 90.1 (C-1’), 100.9 (C-4a), 121.4 (C-6), 149.0 (C-7a), 152.4 (C-2), 156.9 (C-4). HRMS (ESI): calculated for $C_{11}H_{14}BrN_4O_3$ ($[M+H]^+$): 329.0244, found: 329.0240.

4-amino-N7-(3’-deoxy-β-D-ribofuranosyl)-pyrrolo[2,3-*d*]pyrimidine (9)[5] **9** was prepared as described above for **10**. **6** (0.16 g, 0.60 mmol) was converted into **9** (0.065 g, 0.26 mmol) in 43 % yield. ^{1}H NMR (300 MHz, DMSO-d_6) δ: 1.91 (ddd, *J* = 12.9, 6.6, 3.6 Hz, 1H, H-3’’), 2.18 (ddd, *J* = 12.9, 8.4, 6.3 Hz, 1H, H-3’), 3.49 (ddd, *J* = 11.7, 5.7, 4.5 Hz, 1H, H-5’’), 3.62 (ddd, *J* = 11.7, 5.4, 3.6 Hz, 1H, H-5’), 4.24 – 4.29 (m, 1H, H-4’), 4.38 – 4.42 (m, 1H, H-2’), 5.05 (t, *J* = 5.4 Hz, 1H, OH-5’), 5.51 (d, *J* = 4.5 Hz, 1H, OH-2’), 6.00 (d, *J* = 2.7 Hz, 1H, H-1’), 6.56 (d, *J* = 3.6 Hz, 1H, H-5), 6.99 (br. s, 2H, NH_2), 7.32 (d, *J* = 3.6 Hz, 1H, H-6), 8.05 (s, 1H, H-2). HRMS (ESI): calculated for $C_{11}H_{15}N_4O_3$ ($[M+H]^+$): 251.1139, found: 251.1136. Spectral data are in accordance with literature values.[5]

4-amino-5-bromo-N7-(2’-deoxy-β-D-ribofuranosyl)-pyrrolo[2,3-*d*]pyrimidine (12)[7] **11** (0.090 g, 0.36 mmol, 1 eq.) was dissolved in DMF (1.8 mL, 5.0 mL / mmol SM) under argon. Next, a solution of NBS (0.065 g, 0.36 mmol, 1 eq.) in DMF (1.8 mL, 5.0 mL / mmol SM) was added dropwise. After complete addition, the mixture was stirred with the occlusion of light for another 30 minutes at ambient temperature. Then, the solvent was removed *in vacuo* and the residue purified by column chromatography (1 → 10 % MeOH / DCM) to give **12** (0.025 g, 0.076 mmol) as a slight yellow solid in 21 % yield. 1H NMR (300 MHz, DMSO-d6) δ: 2.16 (ddd, *J* = 13.2, 6.0, 2.7 Hz, 1H, H-2’’), 2.41 – 2.45 (m, 1H, H-2’), 3.46 – 3.60 (m, 2H, H-5’, H-5’’), 3.80 – 3.83 (m, 1H, H-4’), 4.30 – 4.35 (m, 1H, H-3’), 5.02 (t, *J* = 5.4 Hz, 1H, OH-5’), 5.25 (d, *J* = 4.2 Hz, 1H, OH-3’), 6.50 (dd, *J* = 8.4, 6.0 Hz, 1H, H-1’), 6.78 (br. s, 2H, NH2), 7.63 (s, 1H, H-6), 8.10 (s, 1H, H-2). Spectral data are in accordance with literature values.[7]

4-amino-N7-(β-D-ribofuranosyl-5’-*O*-monophosphate)-pyrrolo[2,3-*d*]pyrimidine diammonium salt (9-MP) **9** (0.060 g, 0.24 mmol, 1 eq.) was suspended in anhydrous triethylphosphate (2.0 mL, 8.0 mL/mmol SM) under nitrogen, and cooled in an ice-water bath at 0 °C. Then, phosphorusoxychloride (0.022 mL, 0.24 mmol, 1 eq.) was added and the resulting mixture stirred at 0 °C for 2 hours. Then, 0.5 M aqueous ammoniumbicarbonate solution was added slowly and the mixture warmed to ambient

temperature. Purification by column chromatography (i-PrOH/water/sat. aq. NH_3 gradient: 100/0/0 → 20/1/1 → 10/1/1 → 7/1/1 → 6/2/2) gave **9-MP** (0.035 g, 0.11 mmol) as a slight yellow solid in 46 % yield. ^{1}H NMR (300 MHz, DMSO-d_6) δ: 1.95 (ddd, *J* = 12.6, 6.6, 4.2 Hz, 1H, H-3’’), 2.17 – 2.26 (m, 1H, H-3’), 3.70 – 3.82 (m, 2H, H-5’, H-5’’), 4.30 – 4.36 (m, 1H, H-4’), 4.39 – 4.43 (m, 1H, H-2’), 6.05 (d, *J* = 3.0 Hz, 1H, H-1’), 6.57 (d, *J* = 3.6 Hz, 1H, H-5), 6.98 (br. s, 2H, NH_2), 7.37 (d, *J* = 3.6 Hz, 1H, H-6), 8.05 (s, 1H, H-2). ^{31}P NMR (121.5 MHz, DMSO-d_6) δ: -0.46. ^{13}C NMR (75 MHz, DMSO-d_6) δ: 35.5 (C-3’), 65.8 (d, *J* = 6.0 Hz, 1C, C-5’), 74.7 (C-2’), 78.3 (C-4’), 89.5 (C-1’), 99.6 (C-5), 102.6 (C-4a), 121.3 (C-6), 149.9 (C-7a), 151.6 (C-2), 157.4 (C-4). HRMS (ESI): calculated for $C_{11}H_{14}N_4O_6P$ ([M-H]$^-$): 329.0656, found: 329.0666.

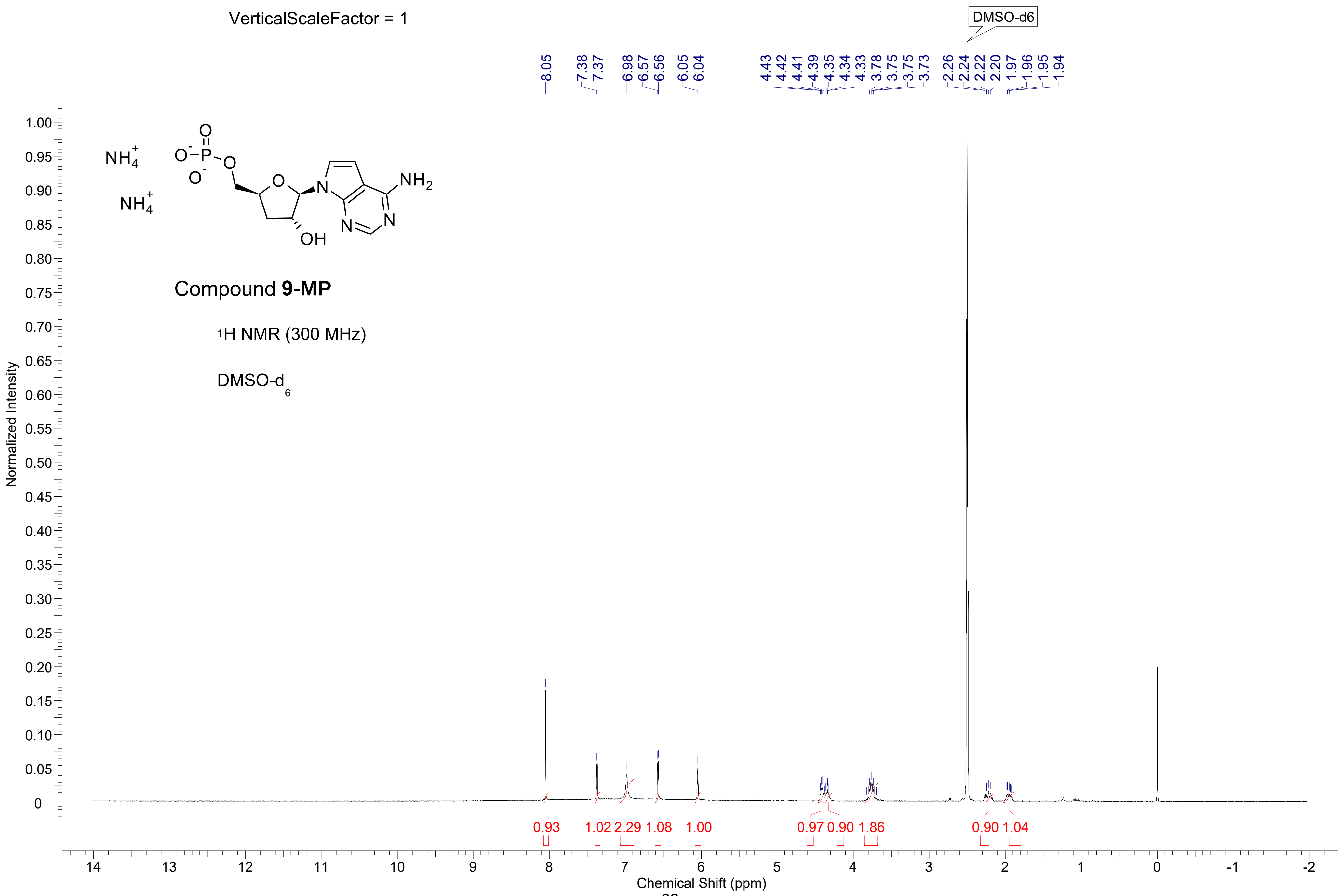
VerticalScaleFactor = 1
DMSO-d6
8.05
7.38
7.37
6.98
6.57
6.56
6.05
6.04
4.43
4.42
4.41
4.39
4.35
4.34
4.33
3.78
3.75
3.75
3.73
2.26
2.24
2.22
2.20
1.97
1.96
1.95
1.94
Compound 9-MP
1H NMR (300 MHz)
DMSO-d6
Normalized Intensity
0.93
1.02
2.29
1.08
1.00
0.97
0.90
1.86
0.90
1.04
Chemical Shift (ppm)

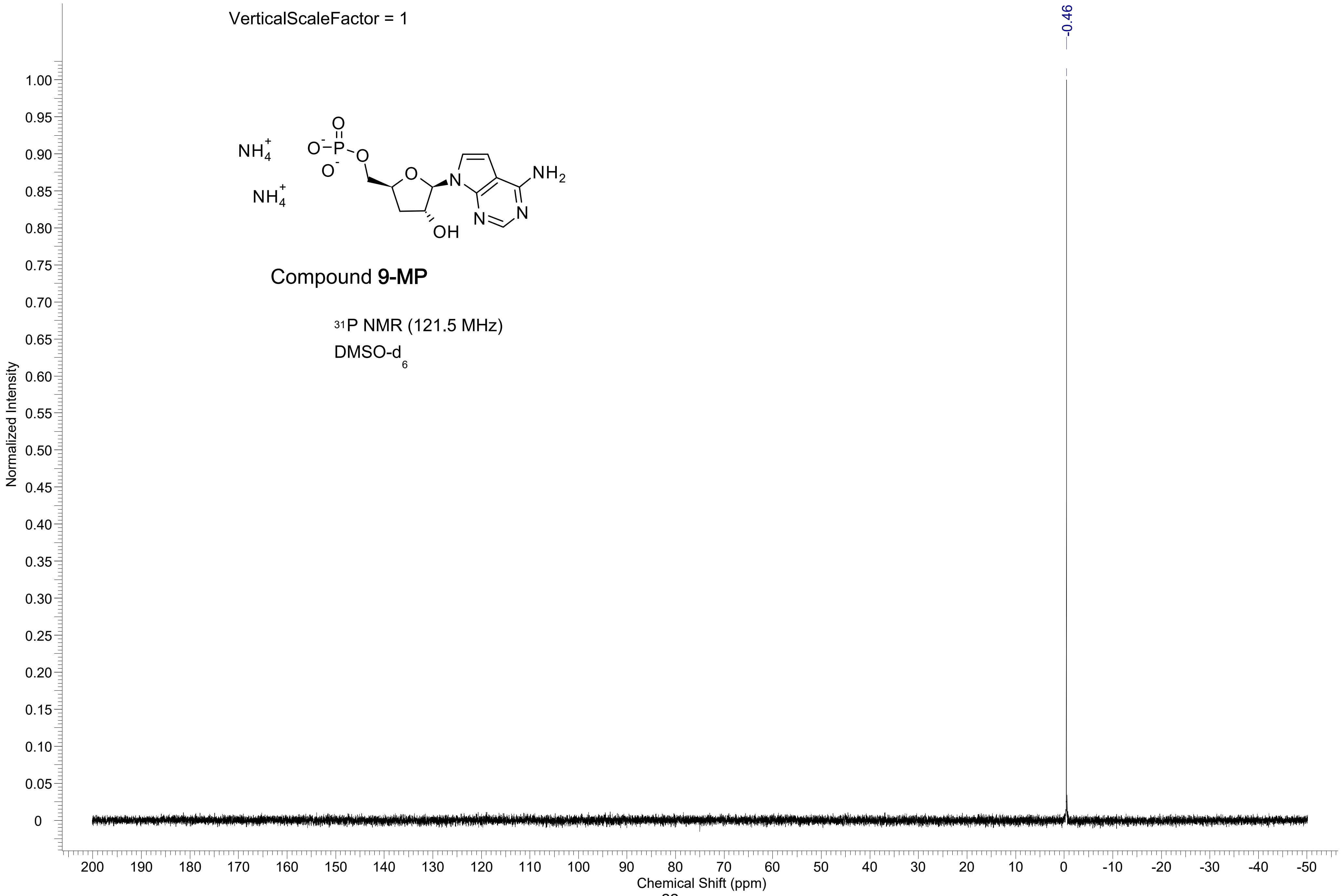
VerticalScaleFactor = 1
-0.46
Compound **9-MP**
31P NMR (121.5 MHz)
DMSO-d6
Normalized Intensity
Chemical Shift (ppm)

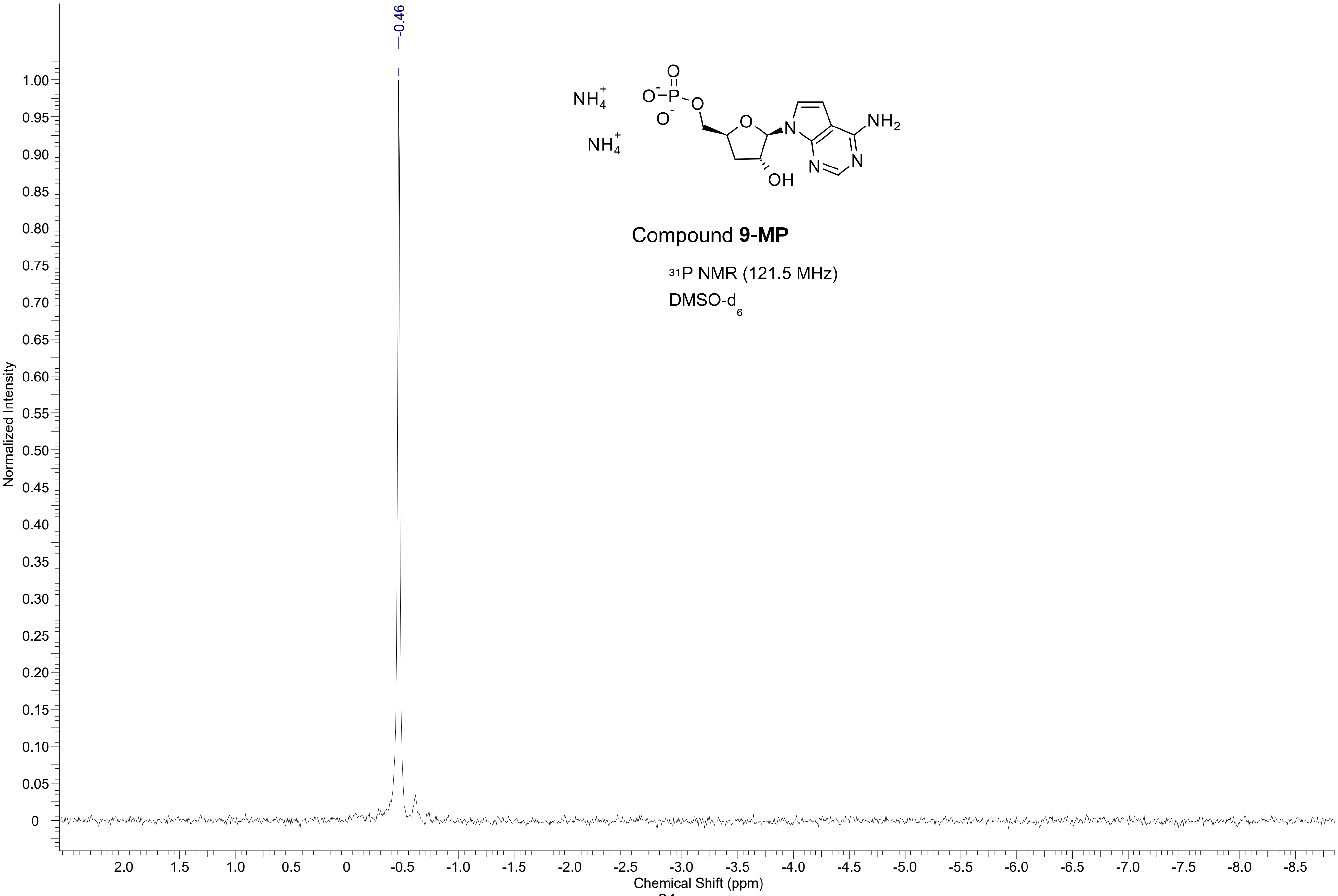

-0.46
NH4+
NH4+
O
O-
P
O-
O
O
N
NH2
N
N
OH
Compound 9-MP
31P NMR (121.5 MHz)
DMSO-d6
Normalized Intensity
Chemical Shift (ppm)

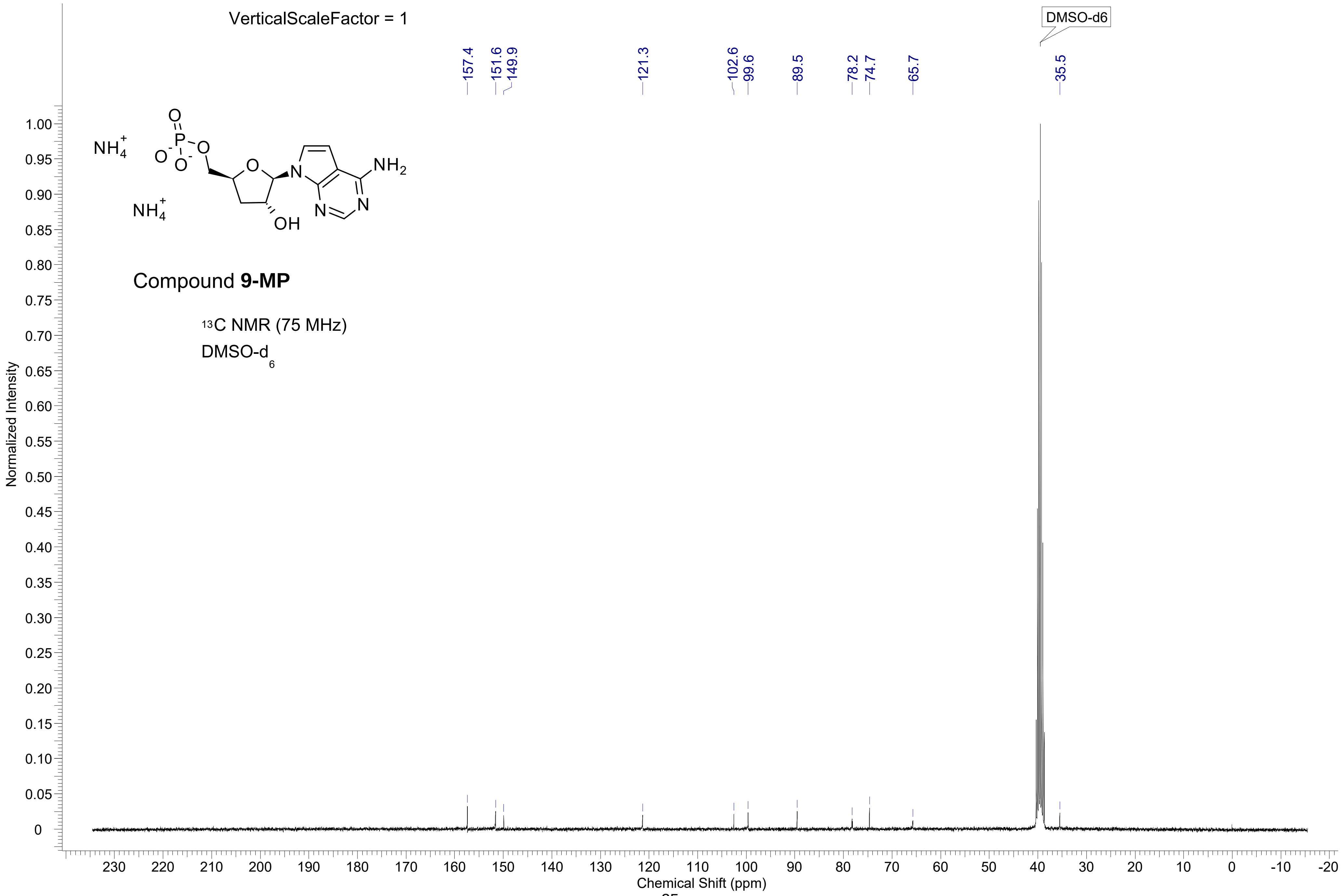
VerticalScaleFactor = 1
DMSO-d6
157.4
151.6
149.9
121.3
102.6
99.6
89.5
78.2
74.7
65.7
35.5
NH4+
NH4+
NH2
OH
Compound 9-MP
13C NMR (75 MHz)
DMSO-d6
Normalized Intensity
Chemical Shift (ppm)

Supplementary references

1 Vodnala, S. K. *et al.* Structure–activity relationships of synthetic cordycepin analogues as experimental therapeutics for African trypanosomiasis. *J. Med. Chem.* **56**, 9861-9873, (2013).

2 Ramasamy, K., Imamura, N., Robins, R. K. & Revankar, G. R. A facile synthesis of tubercidin and related 7-deazapurine nucleosides via the stereospecific sodium salt glycosylation procedure. *Tetrahedron Lett.* **28**, 5107-5110, (1987).

3 Seela, F. & Ming, X. 7-Functionalized 7-deazapurine β-D and β-L-ribonucleosides related to tubercidin and 7-deazainosine: glycosylation of pyrrolo[2,3-*d*]pyrimidines with 1-*O*-acetyl-2,3,5-tri-*O*-benzoyl-β-D or β-L-ribofuranose. *Tetrahedron* **63**, 9850-9861, (2007).

4 Maiti, M. *et al.* Influence of the nucleobase and anchimeric assistance of the carboxyl acid groups in the hydrolysis of amino acid nucleoside phosphoramidates. *Chem. - Eur. J.* **18**, 857-868, (2012).

5 Robins, M. J., McCarthy Jr, J. R., Jones, R. A. & Mengel, R. Nucleic acid related compounds. 5. The transformation of formycin and tubercidin into 2′- and 3′-deoxynucleosides. *Can. J. Chem.* **51**, 1313-1321, (1973).

6 Hulpia, F. *et al.* Discovery of novel 7-aryl 7-deazapurine 3'-deoxy-ribofuranosyl nucleosides with potent activity against *Trypanosoma cruzi*. *J. Med. Chem.* **61**, 9287-9300, (2018).

7 Rosemeyer, H., Tóth, G. & Seela, F. Assignment of anomeric configuration of D-ribo-, arabino-, 2′-deoxyribo-, and 2′,3′-dideoxyribonucleosides by Noe difference spectroscopy. *Nucleosides and Nucleotides* **8**, 587-597, doi:10.1080/07328318908054200 (1989).
