# Supplementary material for: Combining tubercidin and cordycepin scaffolds results in highly active candidates to treat late-stage sleeping sickness
Source: Nat Commun. 2019 Dec 5;10:5564. doi: 10.1038/s41467-019-13522-6 (PMC6895180; doi:10.1038/s41467-019-13522-6)
Supplement: Supplementary file 3 — Reporting Summary [file 41467_2019_13522_MOESM3_ESM.pdf]

nature research

Corresponding author(s): Serge Van Calenbergh
Last updated by author(s): Oct 22, 2019

Reporting Summary

Nature Research wishes to improve the reproducibility of the work that we publish. This form provides structure for consistency and transparency in reporting. For further information on Nature Research policies, see Authors & Referees and the Editorial Policy Checklist.

Statistics

For all statistical analyses, confirm that the following items are present in the figure legend, table legend, main text, or Methods section.

n/a	Confirmed	
☐	☒	The exact sample size (*n*) for each experimental group/condition, given as a discrete number and unit of measurement
☐	☒	A statement on whether measurements were taken from distinct samples or whether the same sample was measured repeatedly
☐	☒	The statistical test(s) used AND whether they are one- or two-sided<br>*Only common tests should be described solely by name; describe more complex techniques in the Methods section.*
☒	☐	A description of all covariates tested
☒	☐	A description of any assumptions or corrections, such as tests of normality and adjustment for multiple comparisons
☐	☒	A full description of the statistical parameters including central tendency (e.g. means) or other basic estimates (e.g. regression coefficient) AND variation (e.g. standard deviation) or associated estimates of uncertainty (e.g. confidence intervals)
☒	☐	For null hypothesis testing, the test statistic (e.g. *F*, *t*, *r*) with confidence intervals, effect sizes, degrees of freedom and *P* value noted<br>*Give P values as exact values whenever suitable.*
☒	☐	For Bayesian analysis, information on the choice of priors and Markov chain Monte Carlo settings
☒	☐	For hierarchical and complex designs, identification of the appropriate level for tests and full reporting of outcomes
☒	☐	Estimates of effect sizes (e.g. Cohen's *d*, Pearson's *r*), indicating how they were calculated

Our web collection on statistics for biologists contains articles on many of the points above.

Software and code

Policy information about availability of computer code

Data collection	Chromperfect 6.0.14 for chromatograms<br>SoftWoRx suite 2.0 for microscopy
Data analysis	Microsoft Excel, Graphpad prism 5.0, 6.01 and 7.04, FlowJo X, BLI images from the IVIS Spectrum In Vivo Imaging System (Perkin Elmer) were analyzed using LivingImage v4.3.1. SoftWoRx suite 2.0 for microscopy (image deconvolution) then processing using Fiji software ((https://imagej.net/Fiji; Schindelin, J., et al. Nat. Methods 2012, 9 (7), 676-682.

For manuscripts utilizing custom algorithms or software that are central to the research but not yet described in published literature, software must be made available to editors/reviewers. We strongly encourage code deposition in a community repository (e.g. GitHub). See the Nature Research guidelines for submitting code & software for further information.

Data

Policy information about availability of data

All manuscripts must include a data availability statement. This statement should provide the following information, where applicable:

- Accession codes, unique identifiers, or web links for publicly available datasets
- A list of figures that have associated raw data
- A description of any restrictions on data availability

Genome sequence and annotation information was obtained from TritrypDB (http://www.tritrypDB.org).

Field-specific reporting

Please select the one below that is the best fit for your research. If you are not sure, read the appropriate sections before making your selection.

☒ Life sciences ☐ Behavioural & social sciences ☐ Ecological, evolutionary & environmental sciences

For a reference copy of the document with all sections, see nature.com/documents/nr-reporting-summary-flat.pdf

Life sciences study design

All studies must disclose on these points even when the disclosure is negative.

Sample size	Animal experimental studies were with a lower number of animals (n= 3-4/group) than calculated by our standard power analysis, suggesting the use of 6 mice/group (2-sample t-test, power= 80%, alpha=0.05). Ethical considerations, combined with experience with these models and a relative low variability have been the basis of justifying these reduced numbers. Observations were confirmed in an independent repeat experiment.
Data exclusions	BLI images of mice that succumbed due to the stage II CNS disease prior to the start of treatment were not included. These data are not relevant to the action of the compound.
Replication	All compound evaluations and in vivo efficacy studies were replicated in at least two independent experiments.
Randomization	Allocation of animals to experimental groups was random.
Blinding	Follow-up of parasitemia and BLI imaging were not conducted in a blinded fashion. Drug administration of the reference drug (topical) and the test compound and vehicle control (oral gavage) were different and could therefore not be blinded.

Reporting for specific materials, systems and methods

We require information from authors about some types of materials, experimental systems and methods used in many studies. Here, indicate whether each material, system or method listed is relevant to your study. If you are not sure if a list item applies to your research, read the appropriate section before selecting a response.

Materials & experimental systems

n/a	Involved in the study	
☒	☐	Antibodies
☐	☒	Eukaryotic cell lines
☒	☐	Palaeontology
☐	☒	Animals and other organisms
☒	☐	Human research participants
☒	☐	Clinical data

Methods

n/a	Involved in the study	
☒	☐	ChIP-seq
☐	☒	Flow cytometry
☒	☐	MRI-based neuroimaging

Eukaryotic cell lines

Policy information about cell lines

Cell line source(s)

MRC-5 SV2 (Sigma-Aldrich / European Collection of Authenticated Cell Cultures)

T. brucei cell lines:

T. brucei brucei Lister 427
T. brucei brucei TbAT1-KO derived from Lister 427: Matovu E, et al.; Eukaryot. Cell. 2003; 2(5):1003–1008.
T. brucei brucei B48 derived from TbAT1-KO (derived from Lister 427): Bridges, D.J., et al., Mol. Pharmacol., 2007. 71(4): p. 1098-1108.
T. brucei brucei ISMR1; Eze, A. A. et al.: PLoS Neglected Trop. Dis. 2016, 10 (8), e0004791.
T. brucei brucei B48 + TbAT1; Munday, J. C., et al.: Mol. Microbiol. 2015, 96 (4), 887-900.

T. brucei brucei Squib 427: Kaiser M, Maes L, Tadoori LP, Spangenberg T, Ioset JR; J. Biomol. Screen. 2015; 20(5), 634-645.
T. brucei rhodesiense STIB-900: Kaminsky R, Brun R; Antimicrob. Agents Chemother. 1998; 42(11):2858–2862.
T. brucei brucei NY-SM: Wirtz, E, Leal, S, Ochatt, C & Cross, G A; Mol. Biochem. Par., 1999; 99, 89–101.
T. brucei brucei BS221: Matovu E, et al.; Eukaryot. Cell. 2003; 2(5):1003–1008.
T. brucei brucei TbAT1-KO derived from T. brucei brucei BS221; Matovu E, Stewart ML, Geiser F, et al.; Eukaryot. Cell. 2003; 2(5):1003–1008.

	T. b. brucei AnTAR1.1 PPYRE9: Van Reet N, Van de Vyver H, Pyana PP, Van der Linden AM, Büscher P; PLoS Negl. Trop. Dis. 2014; 8(8): e3054.
Authentication	No authentication has been conducted after purchase (MRC-5 SV2)
Mycoplasma contamination	Mycoplasma negative
Commonly misidentified lines (See ICLAC register)	n/a

Animals and other organisms

Policy information about studies involving animals; ARRIVE guidelines recommended for reporting animal research

Laboratory animals	Female Swiss Mus musculus were purchased at the age of 7 weeks from the Janvier Labs (France)
Wild animals	No wild animals were used in this study.
Field-collected samples	No field-collected samples were used in this study.
Ethics oversight	Animal experimental work was approved by the ethical committee of the University of Antwerp (UA-ECD 2015-90).

Note that full information on the approval of the study protocol must also be provided in the manuscript.

Flow Cytometry

Plots

Confirm that:

☒ The axis labels state the marker and fluorochrome used (e.g. CD4-FITC).

☒ The axis scales are clearly visible. Include numbers along axes only for bottom left plot of group (a 'group' is an analysis of identical markers).

☒ All plots are contour plots with outliers or pseudocolor plots.

☒ A numerical value for number of cells or percentage (with statistics) is provided.

Methodology

Sample preparation	Culture-derived T. brucei NY-SM cells were exposed to different concentrations of compound 9 for 24 hours. Cells were harvested and washed with PBS before staining with Hoechst 33342 at 5 µg/mL for 25 minutes at 37 °C.
Instrument	MACSQuant flow cytometer (Miltenyi Biotec)
Software	FlowJo X
Cell population abundance	Analyses were performed on pure T. brucei parasite cultures
Gating strategy	Parasites were analyzed within an appropriate FCS/SSC gate, followed by a singlet (SSC-A/SSC-W) gating. The gating strategy is documented in Fig. 7.

☒ Tick this box to confirm that a figure exemplifying the gating strategy is provided in the Supplementary Information.
